# Circular RNA hsa_circ_0007367 promotes the progression of pancreatic ductal adenocarcinoma by sponging miR-6820-3p and upregulating YAP1 expression

**DOI:** 10.1038/s41419-022-05188-8

**Published:** 2022-08-25

**Authors:** Haocheng Zhang, Xiaolei Ma, Luning Wang, Xinyu Li, Di Feng, Meiming Liu, Jiayang Li, Mengxing Cheng, Na Song, Xinxia Yang, Lina Ba, Yating Lei, Ruipu Zhang, Yunxiao Zhu, Wenxiao Xu, Guofen Qiao

**Affiliations:** 1 Department of Pharmacology (State-Province Key Laboratories of Biomedicine-Pharmaceutics of China, Key Laboratory of Cardiovascular Research, Ministry of Education), College of Pharmacy, Harbin Medical University, Harbin, 150086 China; 2 Department of Pharmacy, the Sixth Affiliated Hospital of Harbin Medical University, Harbin, 150086 China; 3 Department of Pathology, Harbin Medical University Cancer Hospital, Harbin, 150086 China; 4 Department of Orthopedics, the Second Affiliated Hospital of Harbin Medical University, Harbin, 150086 China

**Keywords:** Pancreatic cancer, Biomarkers

## Abstract

Circular RNAs (circRNAs) play critical regulatory roles in cancer biological processes. Nevertheless, the contributions and underlying mechanisms of circRNAs to pancreatic ductal adenocarcinoma (PDAC) remain largely unexplored. Dysregulated circRNAs between cancerous tissues and matched adjacent normal tissues were identified by circRNA microarray in PDAC. The biological effect of hsa_circ_007367 both in vitro and in vivo was demonstrated by gain- and loss-of-function experiments. Further, dual-luciferase reporter and RNA pull-down assays were performed to confirm the interaction among hsa_circ_007367, miR-6820-3p, and Yes-associated protein 1 (YAP1). The expression of hsa_circ_007367 and YAP1 were detected by in situ hybridization (ISH) and immunohistochemistry (IHC) using tissue microarray (TMA) in 128 PDAC samples. We first identified that a novel circRNA, hsa_circ_0007367, was markedly upregulated in PDAC tissues and cells. Functionally, in vivo and in vitro data indicated that hsa_circ_0007367 promotes the proliferation and metastasis of PDAC. Mechanistically, we confirmed that hsa_circ_0007367 could facilitate the expression of YAP1, a well-known oncogene, by sponging miR-6820-3p, which function as a tumor suppresser in PDAC cells. The results of ISH and IHC demonstrated that hsa_circ_0007367 and YAP1 were upregulated in PDAC tissues. Furthermore, clinical data showed that higher hsa_circ_0007367 expression was correlated with advanced histological grade and lymph node metastasis in PDAC patients. In conclusion, our findings reveal that hsa_circ_0007367 acts as an oncogene via modulating miR-6820-3p/YAP1 axis to promote the progression of PDAC, and suggest that hsa_circ_0007367 may serve as a potential therapeutic target for treatment of PDAC.

## Introduction

Pancreatic ductal adenocarcinoma (PDAC) is one of the most lethal cancers with the 5-year survival rate of 10% [1] and is the sixth leading cause of cancer death in China [2]. The high mortality of PDAC is attributed to several factors, including rapid invasion, high risk of metastasis, and recurrence. Most PDAC patients are symptomless at early stage until the disease progresses to an advanced phase [3]. Despite the great clinical improvement, the diagnosis and prognosis method for PDAC remain disappointing [4, 5]. Therefore, it is urgent to deeply explore the molecular mechanisms, and identify the potential therapeutic targets of PDAC.

Circular RNAs (circRNAs) are a type of long non-coding RNA (lncRNA) with a covalently closed loop, and originate from pre-mRNA transcripts through the back-splicing [6]. Compared with linear RNAs, circRNAs reveal higher stability due to the circular structure, which without a 5’end or 3’ polyadenylated tails [7]. CircRNAs can derive from introns (intronic circRNA) or exons (exonic circRNA), playing different roles in biological progression [8]. In particular, exonic circRNAs can directly combine with miRNAs to regulate gene expression [9]. Recent years, accumulating evidence has demonstrated that abnormal expression of circRNAs may relate to the development of different diseases, especially in cancers. In PDAC, circBFAR facilitates its progression by acting as a sponge of miR-34b-5p to activate MET signaling [10]; hsa_circ_001653 regulates miR-377/HOXC6 axis to promotes the cell proliferation and metastasis [11]. Based on all the information, circRNAs were considered to be potential biomarkers or therapeutic targets for PDAC. Nevertheless, the crucial molecular mechanism of circRNAs involved in PDAC need further exploration and investigation.

Yes-associated protein 1 (YAP1) is the major downstream effector of the highly conserved Hippo signaling pathway [12]. Hippo signaling pathway is necessary for the control of organ size, tissue homeostasis, and regeneration, and also could regulate YAP1 stability through phosphorylation [13]. Known as a potent oncogene, the abundance and activity of YAP1 are increased in many cancers [14–16]. Meanwhile, YAP1 also induces malignant behavior in PDAC, and it’s imperative to looking for the new molecular regulator for YAP1.

In this study, we identified an oncogenic circRNA, hsa_circ_0007367, through circRNA microarray, which was derived from UBAP2 gene. We first proved its remarkably elevated expression in PDAC tissues and cells. Meanwhile, hsa_circ_0007367 facilitated proliferation, migration, and invasion in vitro, induced tumorigenesis in vivo. Furthermore, hsa_circ_0007367 functioned as a sponge for hsa-miR-6820-3p (miR-6820-3p) to enhance the level of YAP1. Simultaneously, the anti-tumor effects of miR-6820-3p could partly reversed by co-transfection with hsa_circ_0007367. The overexpression of both hsa_circ_0007367 and YAP1 were positively correlated with poorer prognosis of PDAC patients. Our findings indicated hsa_circ_0007367 could be a potential biomarker and provided a new insight into the diagnosis and treatment of PDAC.

## Materials and methods

### Clinical samples and tissue microarray (TMA)

Two independent cohorts were performed in the study: (1) 25 pairs of fresh frozen pancreatic cancer and adjacent nontumorous tissues were acquired the pathology department mentioned above. (2) Patient paraffin samples were collected from the pathology department of Harbin Medical University Cancer Hospital (Harbin, China) from January 2016 to December 2018. Tissue microarray was made of a total of 158 cases were involved 128 cases of PDAC and 30 cases of adjacent normal pancreatic tissues. None of the patients had undergone chemotherapy, radiotherapy, or immunotherapy before surgery. All clinicopathological diagnoses were confirmed by two pathologists. This study was approved by the Ethics Committee of Harbin Medical University (IRB3002720).

### Cell culture and transfection

The human PDAC cell lines AsPC-1, BxPC-3, Capan-1, PANC-1, and SW1990 cells were purchased from Chinese Academy of Sciences (Shanghai, China), and human pancreatic ductal epithelial (HPDE) cells were purchased from Zeye Biotechnology (Shanghai, China). PDAC-1 and BxPC-3 cells were cultured in DMEM (Gibco, China), AsPC-1 and HPDE cells were cultured in RPMI-1640 (Gibco, China), Capan-1 cells were cultured in IMDM (Biosharp, China) and SW1990 cells were cultured in L-15 (Boster, China). The cells authenticated by short tandem repeat (STR) profiling, and tested free from mycoplasma. All of the above complete mediums were supplemented with 10% fetal bovine serum (FBS) (Clark, USA) and 1% penicillin, streptomycin and amphotericin B. All the cells except SW1990 were cultured in a humidified incubator at 37 °C with 100% atmosphere were in a humidified incubator at 37 °C with 5% CO_2_.

All microRNA oligonucleotides including miR-6820-3p mimics or inhibitor and their corresponding controls were purchased from General Biosystem Company (Anhui, China). Small interfering RNA targeting hsa_circ_0007367 (si-hsa_circ_0007367) and the forced expression vector of hsa_circ_0007367 (hsa_circ_0007367) were designed synthesized by RiboBio Biotechnology (Guangzhou, China). Transient transfections were conducted using Lipofectamine 3000 (Invitrogen, USA).

### RNA extraction, reverse transcription, and quantitative real-time PCR analysis (qRT-PCR)

Total RNA was extracted from PDAC cell lines and tissues by using Trizol reagent (Invitrogen, USA). Next, extracted RNA was utilized for reverse transcription to obtain the single-stranded cDNA using the Prime Script RT reagent Kit (Toyobo, Japan) on T100 Thermal Cycler (BIO-RAD, USA). Finally, according to the manufacturer’s instructions, quantitative PCR analyses were performed using a SYBR Green Mix kit (Takara, Japan) to reveal the expression levels of related RNAs, and the process was conducted on the ABI 7500 Real-Time PCR System (Applied Biosystems, USA). The relative fold change of miRNAs circRNAs and mRNAs was calculated using the 2^−△△CT^ method, U6 and β-actin were used as internal references.

### CircRNA microarray analysis

Three pairs of fresh frozen human PDAC tissues and adjacent nontumorous tissues were analyzed by using the Arraystar Human circRNA Array v2 (8 × 15K, Arraystar) (Rockville, USA). Total RNA was digested with RNase R (Epicentre, Inc.) to remove linear RNAs and enrich circular RNAs. Concerned microarray analyses were performed by Aksomic Corporation (Shanghai, China).

### Cell counting Kit-8 (CCK-8) assay

The viability of PDAC cells (PANC-1 and AsPC-1) was assessed by using Cell Counting Kit-8 Kit (CCK-8) assay (Meilunbio, China) following the manufacturer’s protocol. After transfection, cells were seeded in 96-well plates cultured for 0, 24, 48, and 72 h. And then absorbance was measured at 450 nm using a microplate reader (Molecular Devices, USA) after 2 h of incubation at 37 °C.

### 5-Ethynyl-2′-deoxyuridine (EdU) assay

The proliferative function of PANC-1 and AsPC-1 cell lines was examined using the EdU assay kit (Ribobio, China) as the manufacturer’s instructions. The cells used for the experiments were seeded in 24-well plates and incubation with EdU for 2 h, then fixed with 4% paraformaldehyde. The proliferated cells and cell nucleus were stained by using Apollo Dye Solution and the Hoechst33342, respectively. Slices were removed from the 24-well plates, observed and photographed under a fluorescence microscope (Zeiss Axio Scope A1, Germany).

### Wound-healing assay

Cells were cultured and transfected in the 6-well plates with serum-free medium. Next, the changes of wound area between 0 and 24 h were recorded by microscope (Olympus, Japan) at ×100 magnification after using a 10 μl-pipette tip to make.

### Transwell invasion assays

For the transwell invasion assay, the transfected cells were suspended in serum-free medium and 200 μl of the cell suspension was placed in the upper chamber (Corning, USA), containing an 8 μm polycarbonate filter coated with 100 μl of Matrigel (BD, USA). 500 μl culture medium with 10%FBS were added in the lower chamber. 24 h later, the invaded cells on the surfaces of lower chambers were fixed with cold methanol and stained with crystal violet, followed by the counts of cells to invade at ×100 magnification with a Zeiss Axio Scope A1 microscope (Carl Zeiss AG, Germany).

### Tissue immunohistochemistry (IHC)

IHC staining was performed on paraffin-embedded TMA sections. Anti-YAP1 (CST, Danvers, MA, USA) polyclonal antibody at dilution 1:100 was used as primary antibody for IHC staining. Paraffin-embedded tissues were sectioned, dewaxed, hydrated, heated in EDTA (pH 7.2) for antigen retrieval, and inhibited with 3% hydrogen peroxide. The sections were incubated with primary antibodies overnight at 4 °C and the antigen–antibody reactions was developed with a horseradish peroxidase–diaminobenzidine substrate kit (ZSGB-bio, Beijing, China). Images were observed under multifunction microscope (Olympus, Tokyo, Japan) Scoring was performed blindly by two pathologists.

### RNA fluorescence in situ hybridization (FISH)

Cell climbing slices were placed in 24-well plates and cells were seeded in the well before the experiment. Cell density per pore was 60–70% and then pre-hybridized. FISH was performed according to the manufacturer’s protocol with the fluorescent in situ hybridization kit (RiboBio, Guangzhou, China). Briefly, fluorescence-labeled specific probes for hsa_circ_0007367 (RiboBio, Guangzhou, China) were incubated with cells at 37 °C overnight in the dark chamber. A confocal laser scanning microscope (Carl Zeiss AG, Germany) was used to capture the images.

### RNA in situ hybridization (ISH)

Hsa_circ_0007367 expression in PDAC tissues was measured using ISH with biotin-labeled hsa_circ_0007367 by the manufacturer’s instructions. In brief, the specimens on TMA were dewax, rehydrate and digest, followed by hybridizing with the specific hsa_circ_0007367 probe (Geneseed, Guangzhou, China). The samples were incubated with Anti-Digoxin HRP-conjugate (ZSGB-bio, Beijing, China) and stained by DAB (ZSGB-bio, Beijing, China). Then the expression of hsa_circ_0007367 were quantified and imaged in TMAs from PDAC. Scoring was performed blindly by two pathologists.

### RNA pull-down assay

The biotin-labeled hsa_circ_0007367 and control probe were synthesized by Ribobio (Guangzhou, China). First, beads were preprocessed and lysates from cells were collected and incubated with specific hsa_circ_0007367 probes. And then, streptavidin magnetic beads were added and incubated with hsa_circ_0007367 or control probes at 4 °C for 1 h with agitation to pull down the biotin-labeled RNA complex. The RNA-beads complex was treated by RNase R and purified for reverse transcription. The abundance of related microRNAs was analyzed by qRT-PCR.

### Dual-luciferase reporter assay

Wild-type (WT) and mutant (mut) fragments corresponding to the 3′UTR in hsa_circ_0007367 or YAP-1 that may bind to miR-6820-3p were cloned and inserted into the psiCHECK-2 plasmid (Promega). The mimic or NC of miR-6820-3p and hsa_circ_0007367/YAP-1 WT or mut reporter genes were co-transfected into HEK293T cells. After 48 h, the activities of firefly and Renilla luciferase in the cells were detected by luciferase reporter assay system (Promega, Madison, WI, USA).

### Western blot

PANC-1 or AsPC-1 cells were cultured in six-well plates and total protein was extracted by RIPA lysis buffer (Beyotime, China). After the cell lysates were sonicated, and treated with the BCA Protein Analysis kit (Beyotime, China) to detect the concentration of protein by using an enzyme-labeled instrument (Molecular Devices, USA). Proteins were used to SDS-page and transferred to nitrocellulose filter membrane (Pall Corporation, USA), blocked with rapid blocking buffer (GenScript, USA) for 17 min and incubated with primary antibody overnight at 4 °C. The next day it was washed with TBST and incubated with secondary antibodies for 40 min, the membranes were scanned and analyzed with Odyssey infrared imaging instrument (LI-COR, USA). Antibodies used in this study include: anti-β-actin (1:20,000, ABclonal, China), anti-YAP1 (1:1500, Cell Signaling Technology, USA), anti-CTGF (1:1000, Novus Biologicals, USA), and secondary antibody (800R rabbit antibody, 1:1000, LI-COR, USA).

### Animal experiments

The constructs of hsa_circ_0007367 shRNA(sh-hsa_circ_0007367) and control vector (sh-NC) packaged with lentivirus were constructed by Hanbio Biotechnology (Shanghai, China). 5 × 10^7^ PANC-1 cells stably transfected sh-hsa_circ_0007367 or sh-NC in were resuspended in 200 μl high concentrations of matrigel. Ten 4-week-old male BALB/c nude mice (Charles River, Beijing, China) were randomly divided into two groups (*n* = 5/group) and each mouse was injected subcutaneously under armpit with 200 μl cell suspension of sh-hsa_circ_0007367 or sh-NC, testing the tumor volume (length × width^2^/2) in mice every week. Four weeks later, the mice were sacrificed by cervical vertebra detachment and tumor tissues were collected, measured, and weighed. This study was approved by the Ethics Committee of Harbin Medical University.

### Statistical analysis

The statistical analyses were mainly conducted using SPSS 16.0 (IBM, SPSS, Chicago, IL, USA) and GraphPad Prism 8.0 (GraphPad Software Inc., CA, USA). All data are presented as means ± standard deviation (SD). Researchers were blinded to the group allocation both during the experiment and when assessing the outcome. Kaplan–Meier method and log-rank test were used for overall survival (OS) analysis. Correlation analysis between groups was performed by Pearson correlation coefficient. *P* < 0.05 was considered statistically significant.

## Results

### The identification and characteristics of hsa_circ_0007367 in PDAC

**Fig. 1:**
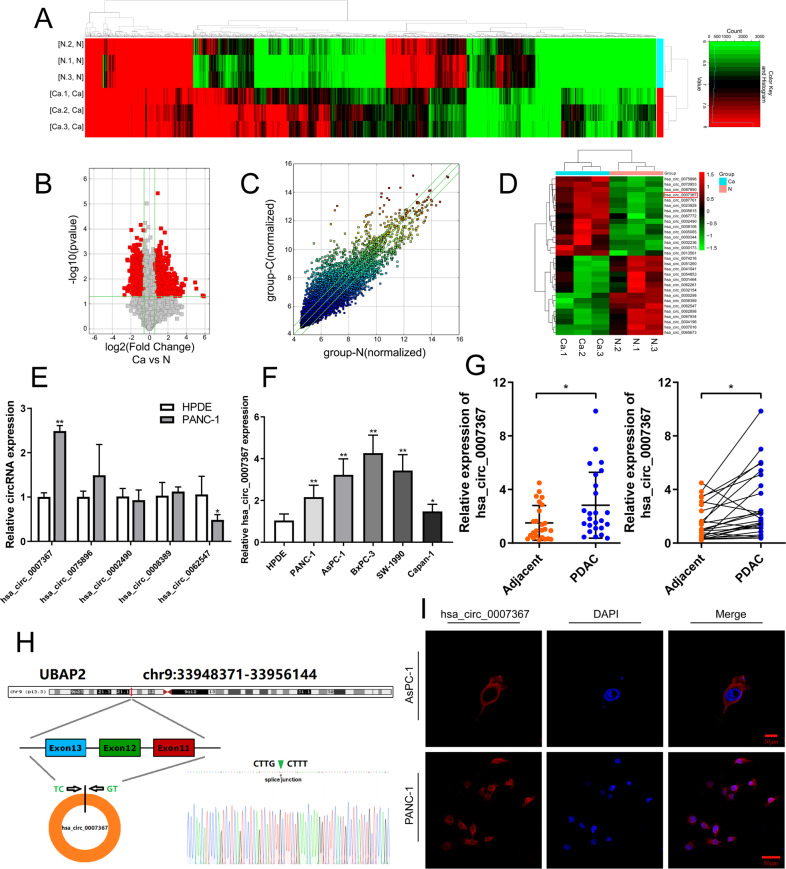
Identification and characterization of hsa_circ_0007367 in PDAC. **A** Hierarchical clustering of differentially expressed circRNAs in 3 pairs of PDAC and their matched normal tissues. Red represents upregulated circRNAs, and green represents downregulated circRNAs. **B** Volcano plot of differentially expressed circRNAs, the −log10 (*p*-value) and the log2 (fold change) are plotted on the y and x axes, respectively. The red dots represent the differentially expressed circRNAs with statistical significance. **C** The scatter plot shows the dysregulated circRNAs in PDAC tissues compared with matched normal pancreas tissues. The space above the top green line and below the bottom green line indicated more than 1.5-fold change. **D** The heatmap for 15 differentially up- and downregulated circRNAs. **E** qRT-PCR analysis of the five dysregulated circRNAs in PANC-1 cells compared to HPDE cells (the normal pancreatic cell line). **F**, **G** qRT-PCR for the expression of hsa_circ_0007367 in PDAC cell lines and tissues. **H** Schematic illustration of the genomic location and splicing pattern of hsa_circ_0007367, with the splicing site identified by Sanger sequencing. **I** FISH assay showed the subcellular localization of hsa_circ_0007367 in PDAC cells. All data are shown as the mean ± SD of at least three independent experiments. *p < 0.05, **p < 0.01.

To identify key circRNAs that involved in PDAC progression, circRNA microarray data was analyzed from 3 pairs of PDAC and matched normal pancreas tissues (Fig. 1A).

There are 13,582 distinct circRNAs detected in total. With the cut-off criteria of fold-change >1.5 or fold-change <0.5 and *P*-value < 0.05, 1753 differentially expressed circRNAs were identified, 988 of which were upregulated and 765 were downregulated (Fig. 1B, C). 15 upregulated and 15 downregulated circRNAs from those differentially expressed were selected to further investigate (Fig. 1D), among them, qRT-PCR results showed that hsa_circ_0007367 was a significantly upregulated circRNAs in both microarray and PDAC cell lines (Fig. 1E, F). Furthermore, qRT-PCR was performed to detect expression levels of hsa_circ_0007367 in 25 pairs of PDAC samples and we found that its expression was higher in cancerous tissues than in paired adjacent normal tissues (Fig. 1G). We then focused on this circRNA for further study.

Based on the circBase annotation [17], hsa_circ_0007367 is derived from exons 11, 12, and 13 of the UBAP2 gene and with a length of 472 bp. Sanger sequencing validated back-splicing junction of hsa_circ_0007367 (Fig. 1H). Subsequently, FISH assays against hsa_circ_0007367 revealed the predominant cytoplasmic distribution of hsa_circ_0007367 in PDAC cells (Fig. 1I). Collectively, our results demonstrated that hsa_circ_0007367 was upregulated in PDAC cell lines and tissues.

### Hsa_circ_0007367 promotes the proliferation, migration, and invasion of PDAC cells in vitro

**Fig. 2:**
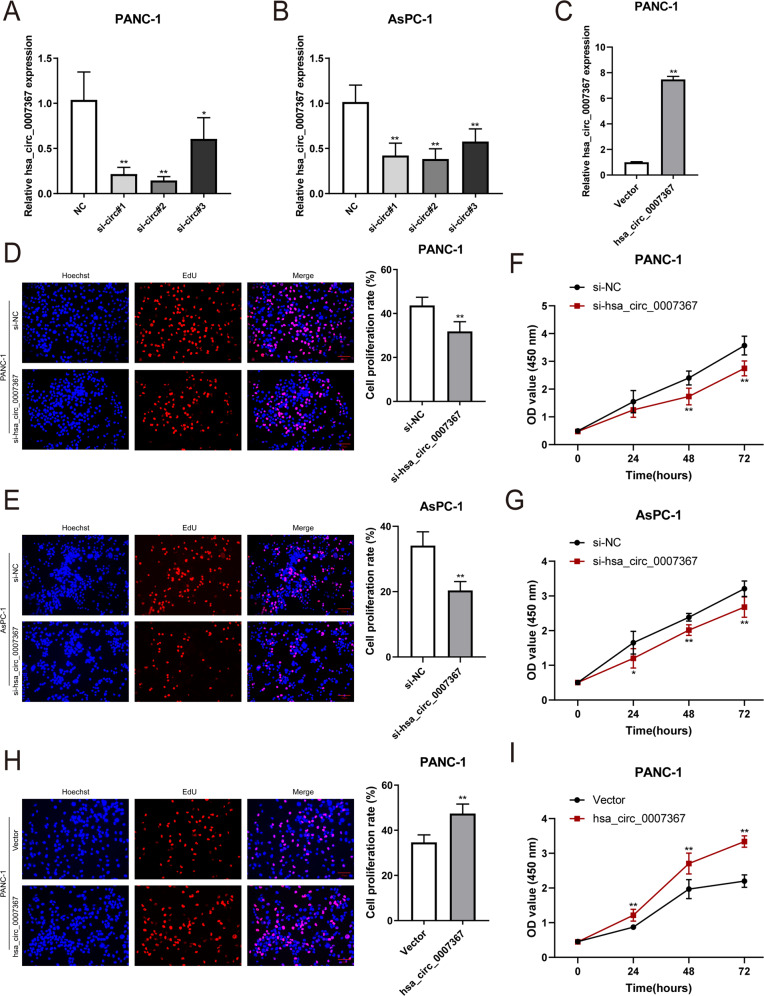
Hsa_circ_0007367 promotes the proliferation of PDAC cells in vitro. **A**–**C** The expression of hsa_circ_0007367 in PANC-1 and AsPC-1 cells was analyzed by qRT-PCR after treated with three siRNAs (**A**, **B**) and transfected with hsa_circ_0007367 plasmid (**C**). **D**–**I** Cell proliferation was detected by EdU (**D**, **E**) and CCK-8 (**F**, **G**) assays in PANC-1 and AsPC-1 cells by knockdown of hsa_circ_0007367, and forced expression hsa_circ_0007367 in PANC-1 cells (**H**, **I**). All data are shown as the mean ± SD of at least three independent experiments. *p < 0.05, **p < 0.01.

**Fig. 3:**
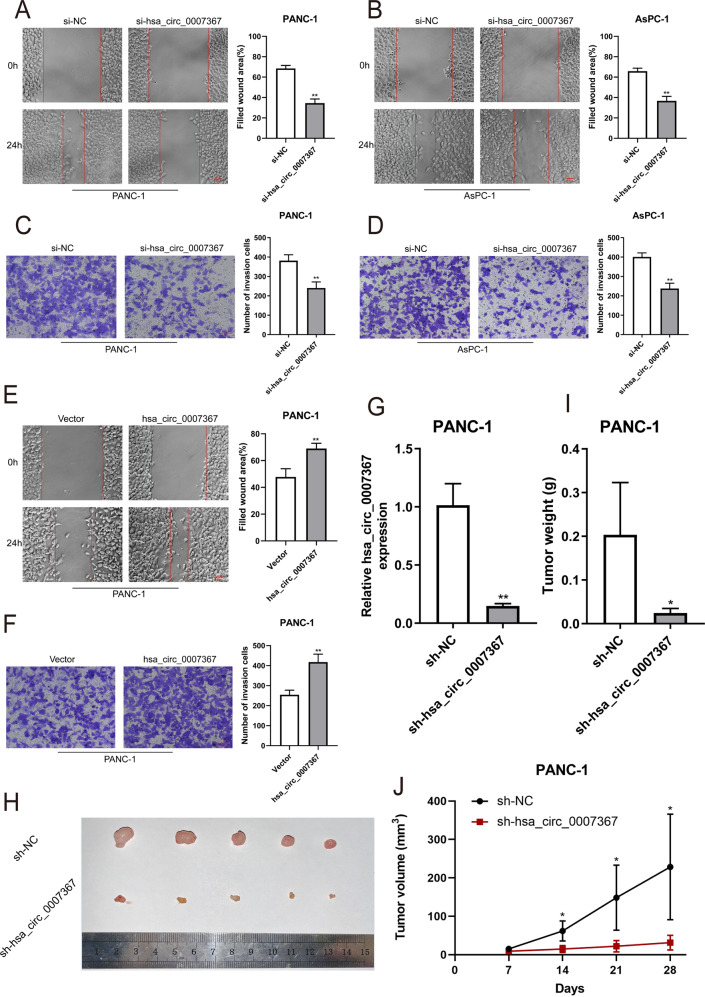
Hsa_circ_0007367 promotes the migration and invasion of PDAC cells in vitro and knockdown of hsa_circ_0007367 inhibits PDAC cell growth in vivo. **A**, **B** The migration was measured by the wound-healing assays in PANC-1 and AsPC-1 cells after knockdown of hsa_circ_0007367. **C**, **D** Transwell invasion assays were performed to verify the invasion capability after knocking down hsa_circ_0007367 in PANC-1 and AsPC-1 cells. **E**, **F** PANC-1 cells transfected with the hsa_circ_0007367 plasmid to detect the migration and invasion capability by wound-healing and transwell invasion assays. **G** Hsa_circ_0007367 knockdown efficiency was detected by qRT-PCR in PANC-1 after transfected with sh-hsa_circ_0007367 and sh-NC plasmid. **H** Representative image of subcutaneous xenograft tumors at day 28 after knockdown of hsa_circ_0007367 compared with sh-NC in PANC-1 cells. **I**, **J** Tumor volume and weight of xenograft after knockdown of hsa_circ_0007367 compared with sh-NC. All data are shown as the mean ± SD of at least three independent experiments. *p < 0.05, **p < 0.01.

To elucidate the biological functions of hsa_circ_0007367 in PDAC, we constructed three siRNAs, which specifically targeted the back-splice site of hsa_circ_0007367. The expression of hsa_circ_0007367 expression was specifically decreased after transfected with siRNAs (Fig. 2A, B). With the most significant downregulation, si-circ#2 was chosen for subsequent study. Meanwhile, cell lines were also transfected with the hsa_circ_0007367 plasmid to overexpress hsa_circ_0007367, and the transfection efficiency was verified by qRT-PCR (Fig. 2C and Supplementary Fig. S1A). EdU and CCK-8 assays showed that the proliferation of PDAC cells were blocked by knockdown of hsa_circ_0007367 (Fig. 2D–G) and hsa_circ_0007367 forced expression elevated the cell viability in PANC-1 and AsPC-1 cells (Fig. 2H, I and Supplementary Fig. S1B, C). In wound healing and transwell invasion assays, we found that hsa_circ_0007367 knockdown suppressed the migration and invasion of PDAC cells (Fig. 3A–D), conversely, forced expression of hsa_circ_0007367 had the opposite effect (Fig. 3E, F and Supplementary Fig. S1D, E). Taken together, these findings indicated that hsa_circ_0007367 performs an oncogenic function in PDAC cells.

### Knockdown of hsa_circ_0007367 suppresses tumor growth from PDAC cells in vivo

To further investigate the potential role of hsa_circ_0007367 in vivo, a xenograft mouse model was constructed. PANC-1 cells were stably transfected with either sh-NC or sh-hsa_circ_0007367 and the knockdown efficiency was analyzed by qRT-PCR (Fig. 3G). Thereafter, these cells were subcutaneously injected into right hind flank of male nude mice. The results showed that hsa_circ_0007367 knockdown inhibited tumor growth (Fig. 3H). Lower tumor volumes and weights were discovered in the sh-hsa_circ_0007367 group compared to the sh-NC group (Fig. 3I, J). Thus, the above results demonstrated that knockdown hsa_circ_0007367 inhibits tumorigenesis of PDAC in vivo.

### Hsa_circ_0007367 functions as a sponge for miR-6820-3p in PDAC

**Fig. 4:**
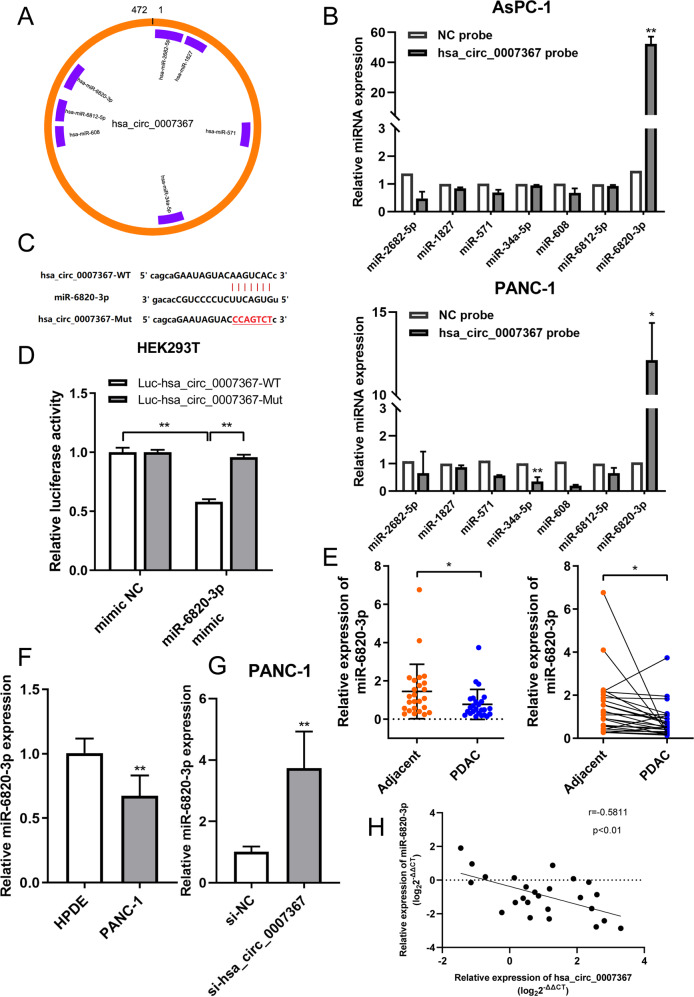
Hsa_circ_0007367 functions as a sponge for miR-6820-3p in PDAC. **A** Schematic drawing showing potential target miRNAs of hsa_circ_0007367 predicted by starbase, circbank, and circular RNA interactome. **B** The relative expression of seven potential target miRNAs in PANC-1 and AsPC-1 cells lysates were detected by qRT-PCR. MiR-6820-3p was stably pulled down by hsa_circ_0007367. **C** Schematic illustration of the wild-type (WT) and mutant (mut) hsa_circ_0007367 luciferase plasmid. **D** The luciferase activities of the hsa_circ_0007367 luciferase reporter vector (WT or mut) in HEK293T cells transfected with miR-6820-3p mimics or mimic NC. **E**, **F** qRT-PCR detected the relative expression of miR-6820-3p in PDAC tissues (**E**) and in HPDE and PANC-1 cell lines (**F**). **G** The relative expression of hsa_circ_0007367 in PANC-1 cells was detected by qRT-PCR after hsa_circ_0007367 knocking down. **H** Correlation analysis between hsa_circ_0007367 and miR-6820-3p expression in PDAC tissues. All data are shown as the mean ± SD of at least three independent experiments. *p < 0.05, **p < 0.01.

Next, we investigated the detailed mechanism of hsa_circ_0007367 in PDAC. Many cytoplasmic circRNAs have been reported to regulate cancer progression by acting as ceRNAs for miRNAs, including lung, stomach, and liver cancer [18–20]. To examined potential miRNAs sponge by hsa_circ_0007367, we performed a cross-analysis using three databases (starbase, circbank, and circular RNA interactome), and seven candidate miRNAs were selected for further study (Fig. 4A). Subsequently, a biotin-labeled hsa_circ_0007367 probe was designed to capture the potential binding partner in RNA pull-down experiments. MiR-6820-3p was highly enriched in sponge complexes detected by qRT-PCR in both PANC-1 and AsPC-1 cells (Fig. 4B).

Accordingly, a dual-luciferase reporter assay was performed to identify the specific binding site between hsa_circ_0007367 and miR-6820-3p. Luciferase reporter plasmids containing wild or mutated hsa_circ_0007367 with the miR-6820-3p binding site were constructed in HEK293T cells (Fig. 4C). The results indicated that transfection of miR-6820-3p mimics markedly suppressed the luciferase activity of the wild-type reporter, whereas mutant construct showed no significant effect (Fig. 4D). However, in contrast to hsa_circ_0007367, miR-6820-3p was downregulated in PANC-1 cells and PDAC tissues (Fig. 4E, F). In addition, hsa_circ_0007367 silencing increased miR-6820-3p levels in PANC-1 cells, as shown using qRT-PCR (Fig. 4G). The results above demonstrate that hsa_circ_0007367 could act as a sponge for miR-6820-3p and suppresses its expression. Furthermore, Pearson correlation analysis indicated that miR-6820-3p expression was negatively associated with hsa_circ_0007367 expression levels, as measured by qRT-PCR in 25 pairs of PDAC tissues (Fig. 4H).

### MiR-6820-3p inhibits the proliferation, migration, and invasion of PDAC cells

**Fig. 5:**
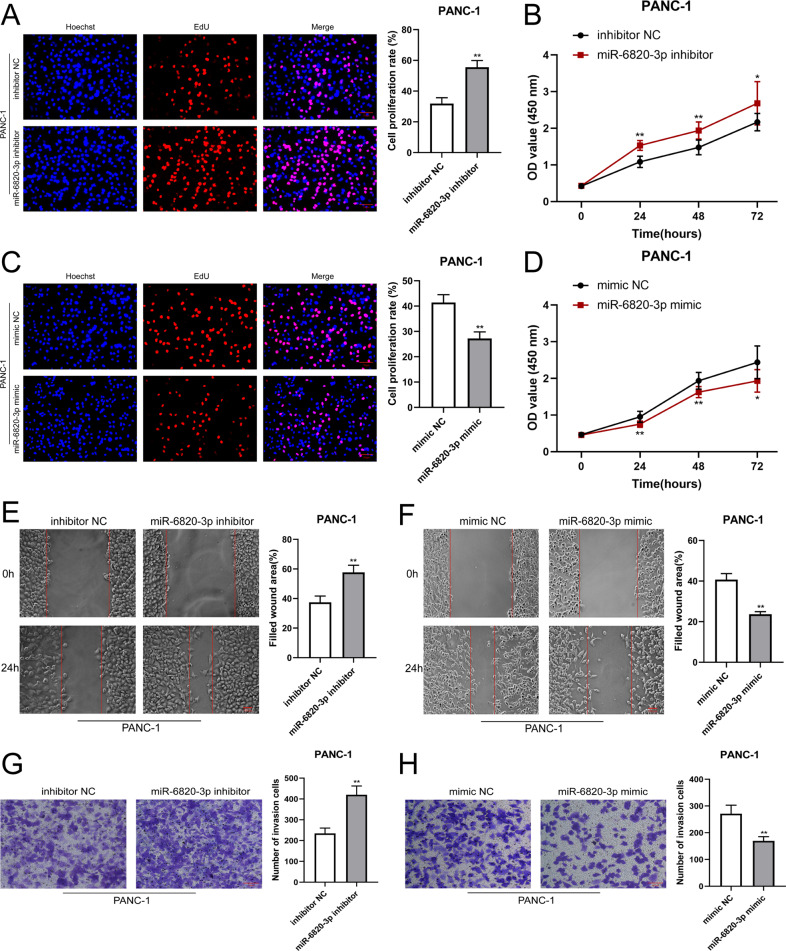
MiR-6820-3p inhibits the proliferation, migration, and invasion of PDAC cells. **A**–**D** EdU (**A**, **C**) and CCK-8 **B**, **D** assay were used to evaluate the proliferation ability of the cells transfected with miR-6820-3p inhibitor or mimics. **E**–**H** Cell migratory and invasive capabilities were determined by wound-healing and transwell invasion assays in PANC-1 cells transfected with miR-6820-3p inhibitor or mimics. All data are shown as the mean ± SD of at least three independent experiments. *p < 0.05, **p < 0.01.

It has been reported that many miRNAs act as anti-cancer role in various tumors [21–23]. Thus, we further assessed the role of miR-6820-3p in PDAC. The EdU and CCK-8 assays showed that downregulated expression of miR-6820-3p promoted PANC-1 cell proliferation (Fig. 5A, B), whereas upregulated expression of miR-6820-3p led to the opposite effects (Fig. 5C, D). A wound-healing assay confirmed that the inhibitor of miR-6820-3p was significantly facilitated cell migration (Fig. 5E), in contrast, miR-6820-3p mimics suppressed cell migration in PANC-1 cells (Fig. 5F). Moreover, the transwell assay indicated that the invasion ability of PANC-1 cells was notably enhanced after transfection with miR-6820-3p inhibitor (Fig. 5G), while the transfection with miR-6820-3p mimics obviously inhibited the PANC-1 cell invasion ability (Fig. 5H). These results demonstrated the importance of miR-6820-3p in suppressing PDAC cell proliferation, migration, and invasion.

### Hsa_circ_0007367 promotes PDAC cell proliferation, migration, and invasion by sponging miR-6820-3p

**Fig. 6:**
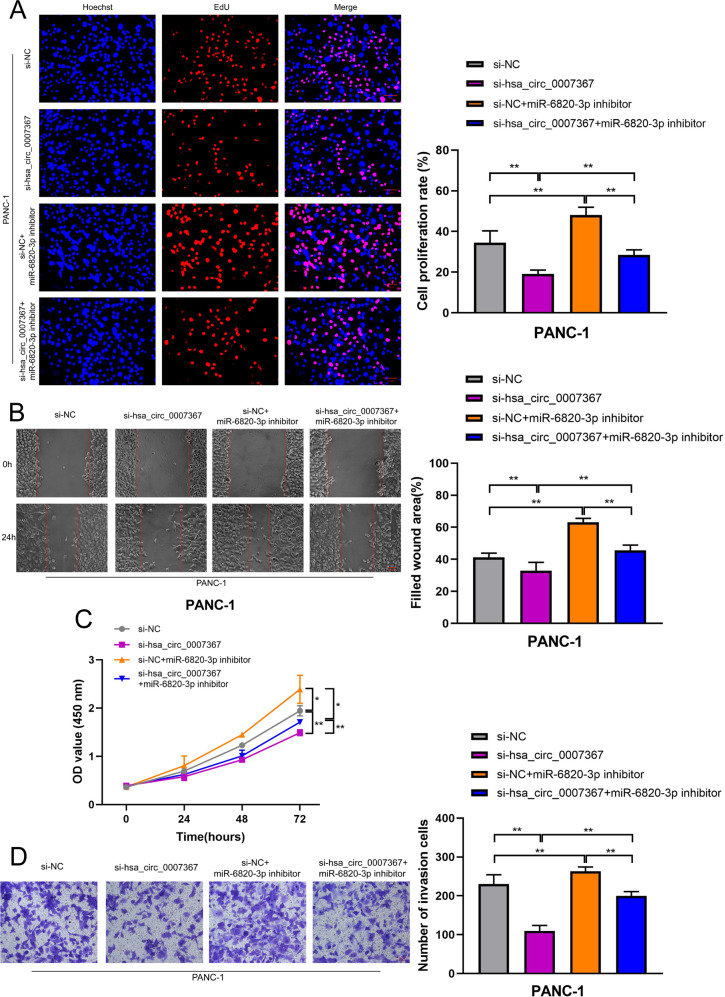
MiR-6820-3p inhibitor reverses the effects of si-hsa_circ_0007367 on proliferation, migration, and invasion in PDAC cells. **A**–**D** EdU (**A**), wound healing (**B**), CCK-8 (**C**), and transwell invasion **D** assays demonstrated that co-transfection with the miR-6820-3p inhibitor could reverse the proliferation, migration and invasion ability of PANC-1 cells after treated with si-hsa_circ_0007367. All data are shown as the mean ± SD of at least three independent experiments. *p < 0.05, **p < 0.01.

Subsequently, we investigated whether hsa_circ_0007367 binding to miR-6820-3p was responsible for the progression of PDAC. As demonstrated by EdU (Fig. 6A) and CCK-8 (Fig. 6C) assays, knockdown of hsa_circ_0007367 inhibited the proliferation ability of PANC-1 cells, while the transfection of the miR-6820-3p inhibitor abolished this effect. Furthermore, hsa_circ_0007367 depletion markedly inhibited the migration and invasion of PANC-1 cells in wound-healing (Fig. 6B) and transwell invasion assays (Fig. 6D), whereas the suppressive effects could be reversed by miR-6820-3p inhibitor. Together, these findings above demonstrated that miR-6820-3p serves a crucial function downstream of hsa_circ_0007367.

### YAP1 is a downstream target of miR-6820-3p and indirectly regulated by hsa_circ_0007367

**Fig. 7:**
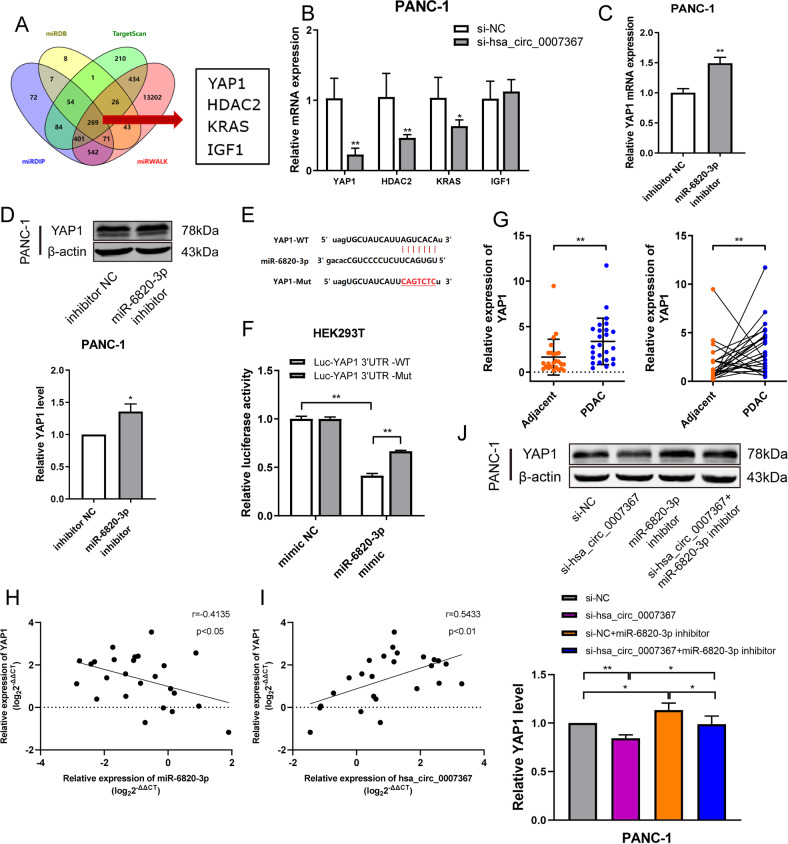
YAP1 is a downstream target of miR-6820-3p and is indirectly regulated by hsa_circ_0007367. **A** Venn diagram showing the potential target genes of miR-6820-3p predicted by miRDB, mirDIP, miRWalk, and TargetScan databases. **B** qRT-PCR analysis of four putative target genes mRNA level in cells transfected with si-hsa_circ_0007367. **C**, **D** YAP1 expression in PDAC cells was detected by qRT-PCR and western blot assays after transfection with the miR-6820-3p inhibitor. **E** Schematic illustrations showed the complementary sequence of miR-6820-3p with the 3′ UTR of YAP1. **F** The luciferase activities of the YAP1 3′ UTR reporter vector (WT or mut) in HEK293T cells transfected with miR-6820-3p mimic. **G** qRT-PCR analysis the expression of YAP1 in PDAC and adjacent tissues. **H**, **I** Correlation analysis between YAP1, hsa_circ_0007367, and miR-6820-3p expression in PDAC tissues. **J** Western blot analysis was performed to measure the protein levels of YAP1. β-actin was used as the loading control. All data are shown as the mean ± SD of at least three independent experiments. *p < 0.05, **p < 0.01.

The potential target genes of miR-6820-3p were predicted by bioinformatics by using miRDB, miRDIP, miRWALK, and TargetScan, and 269 potential target genes were identified (Supplemental table S1). Based on the inhibition of malignant behaviors with miR-6820-3p, the putative target genes with oncogenic function were selected (Fig. 7A). Among them, YAP1 was of particular interest for its low expression in PANC-1 cells treated by si-hsa_circ_0007367 (Fig. 7B). The qRT-PCR and western blot results demonstrated that YAP1 negatively regulated by miR-6820-3p in PANC-1 cells (Fig. 7C, D). Moreover, YAP1 shared the same sequence recognition sites with hsa_circ_0007367 that binds to the “seed” region of miR-6820-3p, suggesting that YAP1 might be the downstream target of hsa_circ_0007367 and miR-6820-3p (Fig. 7E). Therefore, a dual-luciferase reporter assay confirmed that HEK293T cells co-transfected with YAP1-WT and miR-6820-3p mimics exhibited significantly reduced the luciferase activity, which was not dramatically altered in cells co-transfected YAP1-mut and miR-6820-3p mimics (Fig. 7F). Compared with adjacent tissues, YAP1 was upregulated in PDAC tissues (Fig. 7G). Pearson correlation analysis demonstrated YAP1 expression was negatively correlated with miR-6820-3p expression levels, while positively correlated with hsa_circ_0007367 (Fig. 7H, I). Functional experiment results indicated that the effects of overexpression hsa_circ_0007367 could be reversed by co-transfection with si-YAP1 (Supplementary Fig. S2A–D). Meanwhile, western blotting showed that knockdown of hsa_circ_0007367 attenuated the expression of YAP1, whereas the effect was abolished by co-transfection with miR-6820-3p inhibitor (Fig. 7J). The results presented herein indicated that YAP1 was a downstream target of miR-6820-3p, and hsa_circ_007367 may perform its oncogenic biological functions by regulate expression of YAP1 via suppressing miR-6820-3p.

### Upregulated hsa_circ_0007367 expression indicates aggressive clinicopathological characteristics in PDAC patients

**Fig. 8:**
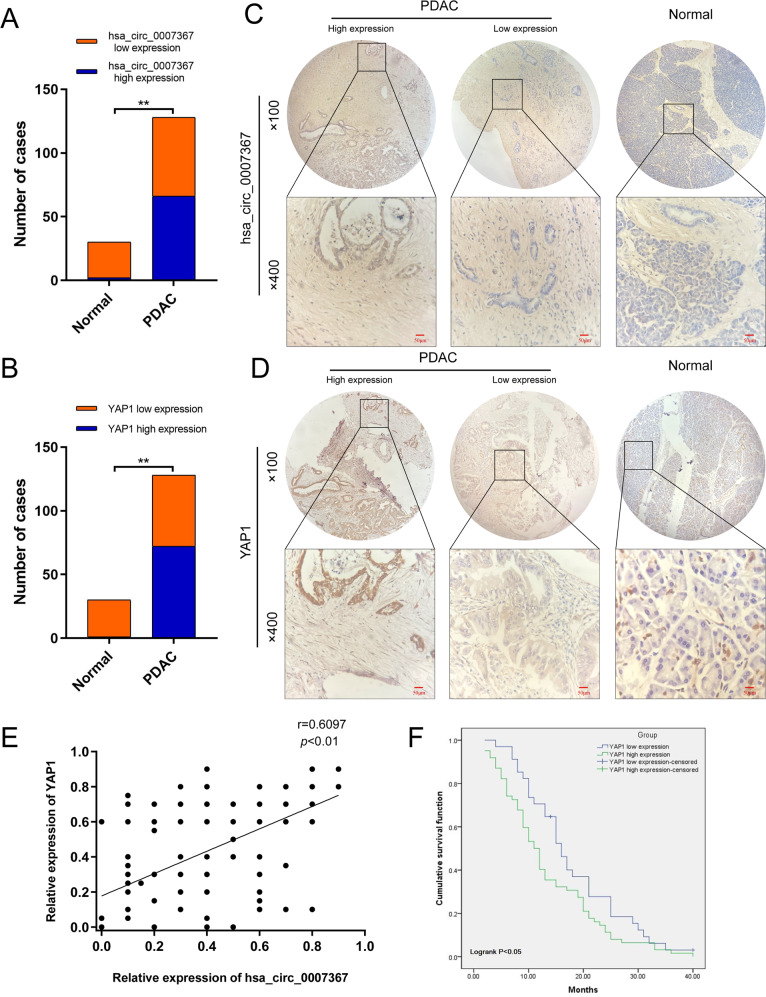
Upregulated hsa_circ_0007367 related with poor prognosis of PDAC patients. **A**, **B** The numbers of hsa_circ_0007367 (**A**) and YAP1 **B** expression in TMAs. **C**, **D** Representative images showed the expression of hsa_circ_0007367 detected by ISH (**C**) and YAP1 detected by IHC (**D**) in TMAs. **E** The correlation between hsa_circ_0007367 and YAP1 expression in TMAs. **F** Kaplan–Meier survival curve for the overall survival of PDAC patients according to the relative expression of YAP1 in TMAs.

To further elucidate the clinical relevance of hsa_circ_0007367 in PDAC patients, we used another cohort of 128 PDAC tissues and 30 normal tissues on TMAs to detect the expression of hsa_circ_0007367 with ISH. The results indicated that there were 66 high hsa_circ_0007367 expression in 128 PDAC samples and only 2 high hsa_circ_0007367 expression in 30 normal samples (Fig. 8A), the expression of hsa_circ_0007367 was dramatically higher in PDAC tissues than in adjacent normal tissues (Fig. 8B). The correlation between hsa_circ_0007367 expression and clinicopathological characteristics of the PDAC patients were listed in Table 1. It displayed that the expression of hsa_circ_0007367 was positively correlated with histological grade and lymph node metastasis. Given that the expression of hsa_circ_0007367 is positively correlated with the mRNA of YAP1 in PDAC fresh tissues in previous study (Fig. 7I), IHC was performed on TMAs to further investigate the expression of YAP1. As shown in Fig. 8C, D, 72 of 128 PDAC samples showed high expression of YAP1, and only 1 showed YAP1 high expression in 30 normal samples. YAP1 was remarkably upregulated and significantly correlated with hsa_circ_0007367 in TMAs (Fig. 8E). In addition, analysis of the clinical characteristics of PDAC patients revealed that YAP1 expression was increased with advanced histological grade and lymph node metastasis (Table 2). Notably, Kaplan–Meier survival analysis showed that there was a positive association between high expression of YAP1 and poor overall survival (*P* < 0.05) (Fig. 8F).

**Table 1:** Correlation between hsa_circ_0007367 expression and clinicopathological characteristics of PDAC patients (*N* = 128).

Characteristics	Total number	Low expression	High expression	*χ*^2^	*P* Value
Age					
<55	44	20	24	0.239	0.711
≥55	84	42	42		
Gender					
Male	76	33	43	1.885	0.208
Female	52	29	23		
Tumor size					
≤2	21	10	11	0.007	1
>2	107	52	55		
Histological grade					
I+II	59	38	21	11.176	0.001**
III	69	24	45		
Lymph node metastasis					
Yes	43	14	29	6.537	0.015*
No	85	48	37		

Chi-square test. *p < 0.05, **p < 0.01.

**Table 2:** Correlation between YAP1 expression and clinicopathological characteristics of PDAC patients (*N* = 128).

Characteristics	Total number	Low expression	High expression	*χ*^2^	*P* Value
Age					
<55	44	21	23	0.431	0.575
≥55	84	35	49		
Gender					
Male	76	32	44	0.206	0.718
Female	52	24	28		
Tumor size					
≤2	21	11	10	0.76	0.472
>2	107	45	62		
Histological grade					
I+II	59	38	21	18.977	<0.001**
III	69	18	51		
Lymph node metastasis					
Yes	43	11	32	8.686	0.004**
No	85	45	40		

Chi-square test. **p < 0.01.

Collectively, these clinical data suggested hsa_circ_0007367 might act as a potential biomarker to predicting the prognosis of PDAC patients.

## Discussion

As novel non-coding RNAs, accumulating evidence indicates that circRNAs involved in pleiotropically modulated cellular function and played important regulatory roles in various tumors [7, 24]. For instance, several circRNAs could function as miRNA sponges, encode polypeptides, interact with proteins and regulate transcription [25]. Based on their highly stable nature and notable tissue specificity, circRNAs will emerge as reliable biomarkers of disease diagnosis and treatment [26]. Although intense studies confirmed the roles of circRNAs in tumor progression, only a few studies have investigated the relationship between circRNAs and PDAC. A previous report demonstrates that CircEYA3 promotes PDAC progression by inducing energy production through the miR-1294/c-Myc axis [27]. Another study reported that circFOKX2 contributed to tumor progression in PDAC by sponging miR-942 and interacting with YBX1 and hnRNPK [28]. In this study, we profiled circRNAs expressions in 3 pairs of PDAC and matched normal pancreas tissues using circRNA microarray analysis, and identified hsa_circ_0007367 was highly expressed both in tissues and cells of PDAC. Therefore, our investigation focused on the potential role of hsa_circ_0007367 in PDAC. Loss- and gain-of-function experiments demonstrated that hsa_circ_0007367 promoted the malignant behavior of PDAC cells in vitro. Besides, we also found knockdown hsa_circ_0007367 suppressed tumor growth in vivo, indicating its oncogenic role in PDAC.

Numerous recent investigations have revealed that CircRNAs primarily function as miRNA sponges in many tumors, including lung adenocarcinoma [29], hepatocellular carcinoma [30], gastric cancer [31], and breast cancer [32].

In the present study, as we know that hsa_circ_0007367 is generated from the coding exons of UBAP2 and localized predominantly in the cytoplasm, we speculated that hsa_circ_0007367 affects downstream target genes by acting as miRNA sponge. Accordingly, we applied three databases (starbase, circbank, and circular RNA interactome) to predict the potential targets of hsa_circ_0007367 and identified 7 miRNAs as candidate targets. Notably, RNA pull-down assays validated that hsa_circ_0007367 interacted with miR-6820-3p. Dual-luciferase reporter assays confirmed the sponge effect and binding sites of hsa_circ_0007367 on miR-6820-3p. Statistical analysis demonstrated that the expression of miR-6820-3p was negatively correlated with hsa_circ_0007367 in PDAC tissues. Abnormal expression of miRNAs is involved in progression of different cancers [33]. Due to the unclear levels and functions of miR-6820-3p in tumors, we detected the expression levels and functions of miR-6820-3p in PDAC. We first demonstrated that miR-6820-3p was downregulated in PDAC tissues and the results of gain- and loss-of-function experiments suggested that miR-6820-3p inhibited the proliferation, migration and invasion of PDAC cells in vitro, implying that miR-6820-3p may act as a tumor suppressor. In addition, further rescue experiments in vitro showed that miR-6820-3p reversed the oncogenic effects of hsa_circ_0007367 in PDAC. Our results indicated that hsa_circ_0007367 plays oncogenic role by directly binding to miR-6820-3p in PDAC.

**Fig. 9:**
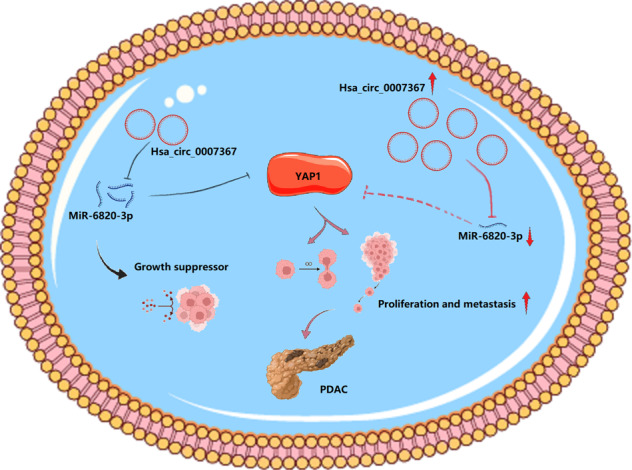
Hsa_circ_0007367/miR-6820-3p/YAP1 axis promoted the progression of PDAC. Hsa_circ_0007367 upregulated YAP1 expression via sponging miR-6820-3p to facilitate the proliferation, migration and invasion of PDAC cells.

In human cancer, miRNAs are often dysregulated and circRNA–microRNA code interferes the regulation of gene expression to impact the progression of disease [34]. We further conducted bioinformatic, qRT-PCR, and western blot assays to detect the downstream target gene of miR-6820-3p. The results showed YAP1 is the most likely target of miR-6820-3p. Another dual-luciferase assay confirmed that miR-6820-3p directly bound to YAP1 to downregulate its expression. YAP1 is a transcriptional coactivator and involved in controlling normal tissue growth and tumor development [35]. Its activity is regulated by the upstream Hippo pathway, which is a highly conserved cell signaling pathway and plays an important role in regulating cell proliferation, metastasis, and tumorigenesis in cancers [36, 37]. Previous studies have reported YAP1’s oncogenic role. Upregulation of YAP1 expression has been detected in breast cancer [38], melanoma [39], and hepatocellular carcinoma [40], suggesting that YAP1 is essential for tumor initiation. Moreover, it was reported that blockaded YAP1 can inhibit cell proliferation and relieve immune suppression within the tumor microenvironment in PDAC [41]. Another research has revealed that YAP1 has the potential to be identified as an outcome predictor, even a therapy target by pharmacological treatment or genetic knockdown in PDAC [42]. There was also a study which documented that in acinar cells, the activation of YAP1 upregulated JAK-STAT3 signaling to promote development of PCAC in mice [43]. Consistent with these findings, the clinical statistics showed that high expression of hsa_circ_0007367 and YAP1 were significantly correlated with aggressive clinicopathological characteristics and poor prognosis in PDAC patients. Given the particular importance of YAP1 in PDAC progression [41, 42], our findings demonstrated that hsa_circ_0007367 acted as a hunter to capture miR-6820-3p in order to alleviate the suppression of its downstream target YAP1, which can promote the progression of PDAC (Fig. 9).

However, our study has several limitations. First, whether hsa_circ_0007367 influences PDAC development via other biological processes, such as interacting with proteins or encoding polypeptides. Second, we validated the expression of hsa_circ_0007367 in PDAC tissues and cells, additionally, it needs to be detected in a wider range of clinical samples, including blood, saliva, and urine, to enhance its diagnostic and therapeutic value. Thus, mechanistic research should be further performed, and more clinical samples to be collected in future study.

## Conclusion

In conclusion, we proved hsa_circ_0007367 was highly expressed in PDAC tissues and cells, and promoted cell proliferation, migration, and invasion in PDAC cells by serving as a sponge for miR-6820-3p. MiR-6820-3p effected as a tumor suppressor to decrease YAP1 expression in PDAC. Clinically, hsa_circ_0007367 expression was positively associated with aggressive clinicopathological characteristics in PDAC patients. Therefore, our findings demonstrated that hsa_circ_0007367 upregulated YAP1 expression via sponging miR-6820-3p to promote PDAC progression, and indicated hsa_circ_0007367 could be a potential biomarker to provide a novel therapeutic strategy in PDAC.

## Supplementary information

Supplemental Figure legend

Supplementary Fig. S1

Supplementary Fig. S2

Supplemental table S1

Supplemental table S2

Supplemental table S3

Original Data File

aj-checklist

## Data Availability

The data generated or analyzed in the current study are available from the corresponding author upon reasonable request.
