# Supplementary material for: Circular RNA hsa_circ_0007367 promotes the progression of pancreatic ductal adenocarcinoma by sponging miR-6820-3p and upregulating YAP1 expression
Source: Cell Death Dis. 2022 Aug 25;13(8):736. doi: 10.1038/s41419-022-05188-8 (PMC9411600; doi:10.1038/s41419-022-05188-8)
Supplement: Supplementary file 1 — Supplemental Figure legend [file 41419_2022_5188_MOESM1_ESM.docx]

**Circular RNA hsa_circ_0007367 promotes the progression of pancreatic ductal adenocarcinoma by sponging miR-6820-3p and upregulating YAP1 expression**

**Haocheng Zhang ^1,2^, Xiaolei Ma^1^, Luning Wang^1^, Xinyu Li^1^, Di Feng^3^, Meiming Liu^1^, Jiayang Li^1^, Mengxing Cheng^1^, Na Song^1^, Xinxia Yang^1^, Lina Ba^1^, Yating Lei^1^,** **Yunxiao Zhu^3^, Wenxiao Xu^1,4^**^#^ **and Guofen Qiao^1^**^#^

^1^Department of Pharmacology (State-Province Key Laboratories of Biomedicine-Pharmaceutics of China, Key Laboratory of Cardiovascular Research, Ministry of Education), College of Pharmacy, Harbin Medical University, Harbin 150086, China.

^2^Department of Pharmacy, the Sixth Affiliated Hospital of Harbin Medical University, Harbin 150086, China.

^3^Department of Pathology, Harbin Medical University Cancer Hospital, Harbin 150086, China.

^4^Department of Orthopedics, the Second Affiliated Hospital of Harbin Medical University, Harbin 150086, China.

^#^Corresponding authors:

Guofen Qiao (qiaogf@hrbmu.edu.cn) and Wenxiao Xu (wenxuhmu@outlook.com)

**Figure legends**

**Supplementary Fig. S1 Overexpression hsa_circ_0007367 promotes the proliferation of AsPC-1 cells in vitro.**

(**A)** The expression of hsa_circ_0007367 was assessed by qRT-PCR in AsPC-1 cells treated with hsa_circ_0007367 plasmid.

**(B and C)** Cell proliferation was detected by EdU (**B**) and CCK-8(**C**) assays in AsPC-1 cells by overexpression hsa_circ_0007367.

(**D)** The migration was measured by the wound healing assays in AsPC-1 cells after overexpression hsa_circ_0007367.

(**E)** Transwell invasion assays were performed to verify the invasion capability after overexpression hsa_circ_0007367 in AsPC-1 cells.

**Supplementary Fig. S2 The effects of overexpression hsa_circ_0007367 could be reversed by co-transfection with si-YAP1.**

**(A-D)** EdU (**A**), wound healing (**B**), CCK-8 (**C**) and transwell invasion(**D**) assays demonstrated that co-transfection with the hsa_circ_0007367 could reverse the proliferation, migration and invasion ability of PANC-1 cells after treated with si-YAP1.

**Supplementary table 1. 269 common elements in miRDIP, miRDB, miRWalk and TargetScan.**

**Supplementary table 2. The sequences of primers used in this study.**

**Supplementary table 3. The sequences of oligonucleotides and probes used in this study.**
