# Supplementary figures and images for: Circular RNA hsa_circ_0007367 promotes the progression of pancreatic ductal adenocarcinoma by sponging miR-6820-3p and upregulating YAP1 expression
Source: Cell Death Dis. 2022 Aug 25;13(8):736. doi: 10.1038/s41419-022-05188-8 (PMC9411600; doi:10.1038/s41419-022-05188-8)

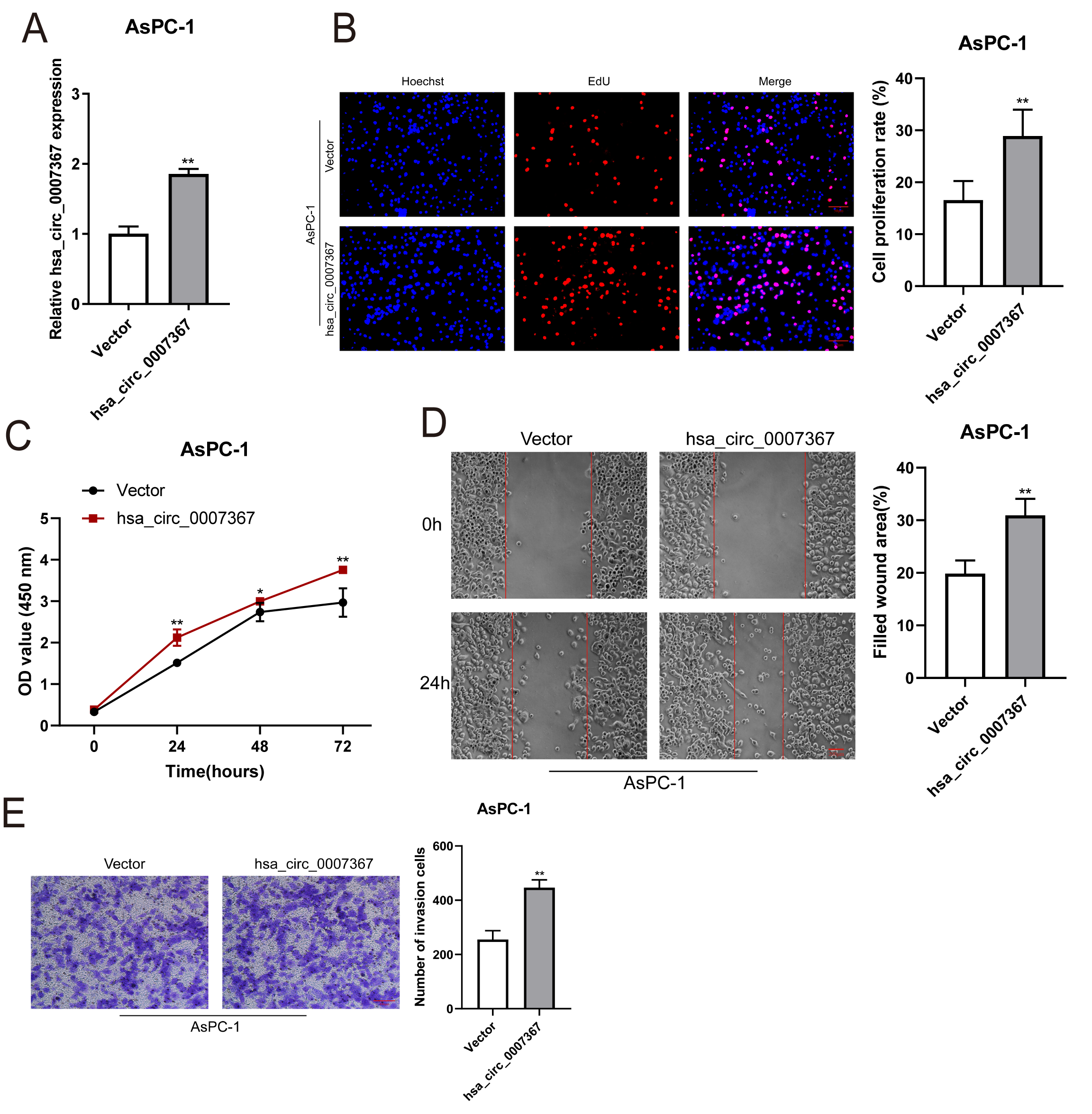

Supplement: Supplementary file 2 — Supplementary Fig. S1 [file 41419_2022_5188_MOESM2_ESM.tif]

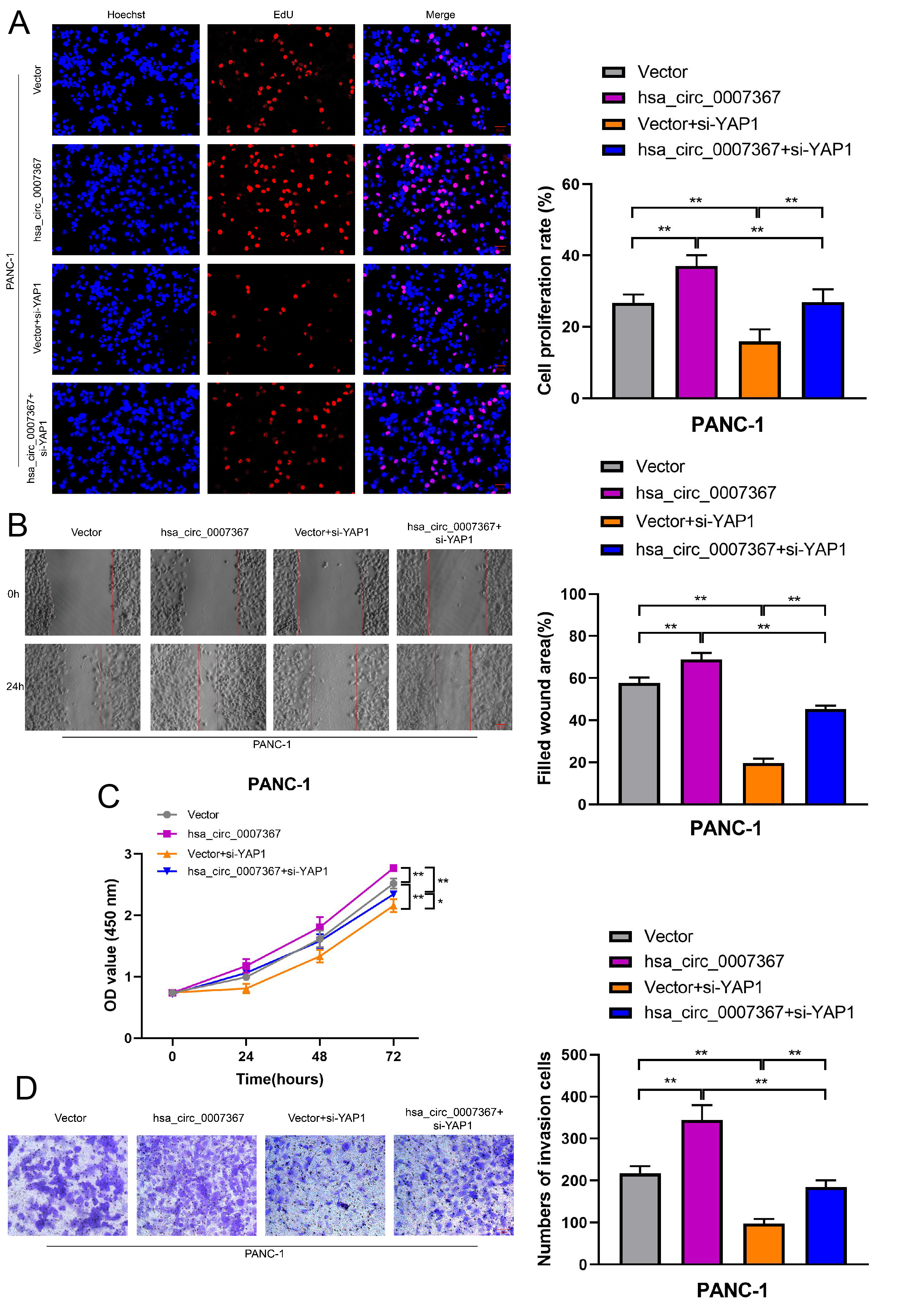

Supplement: Supplementary file 3 — Supplementary Fig. S2 [file 41419_2022_5188_MOESM3_ESM.jpg]

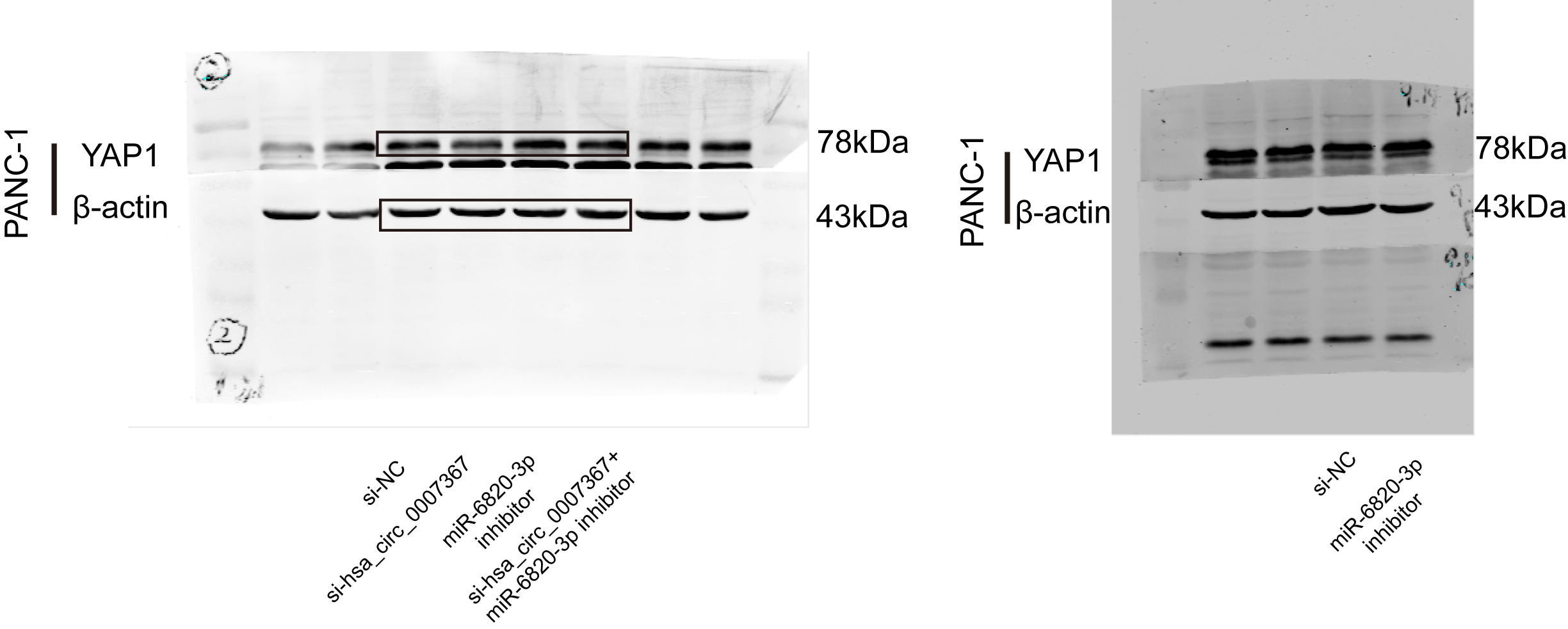

Supplement: Supplementary file 7 — Original Data File [file 41419_2022_5188_MOESM7_ESM.tif]
