# Supplementary material for: Circular RNA hsa_circ_0007367 promotes the progression of pancreatic ductal adenocarcinoma by sponging miR-6820-3p and upregulating YAP1 expression
Source: Cell Death Dis. 2022 Aug 25;13(8):736. doi: 10.1038/s41419-022-05188-8 (PMC9411600; doi:10.1038/s41419-022-05188-8)
Supplement: Supplementary file 5 — Supplemental table S2 [file 41419_2022_5188_MOESM5_ESM.docx]

**Supplement Table 2. The sequences of primers used in this study.**

| **Gene** | **Sequence (5’-3’)** |
| --- | --- |

| hsa_circ_0007367 | forward, CTCAGTCCTCAGTCATCTTGCTTTC  reverse, AGTTGGCTTCTGAGGCTTGAC |  |
| --- | --- | --- |
| β-actin | forward, GCGGACTATGACTTAGTTGCGTTACA  reverse, TGCTGTCACCTTCACCGTTCCA |  |
| hsa-miR-6820-3p | forward, TGTGACTTCTCCCCTGCCACA  reverse, ATCCAGTGCAGGGTCCGAGG | |
| U6 | forward, GCTTCGGCAGCACATATACTAAAAT  reverse, CGCTTCACGAATTTGCGTGTCAT |  |
| YAP1 | forward, TAGCCCTGCGTAGCCAGTTA  reverse, TCATGCTTAGTCCACTGTCTGT |  |
