# Supplementary material for: Circular RNA hsa_circ_0007367 promotes the progression of pancreatic ductal adenocarcinoma by sponging miR-6820-3p and upregulating YAP1 expression
Source: Cell Death Dis. 2022 Aug 25;13(8):736. doi: 10.1038/s41419-022-05188-8 (PMC9411600; doi:10.1038/s41419-022-05188-8)
Supplement: Supplementary file 6 — Supplemental table S3 [file 41419_2022_5188_MOESM6_ESM.docx]

| **siRNAs** | |
| --- | --- |
| si-circRNA#1 sense | CTCAGTCATCTTGCTTTCT |
| si-circRNA#2 sense | CAGTCATCTTGCTTTCTGA |
| si-circRNA#3 sense | CATCTTGCTTTCTGAAACA |
| **miR-6820-3p mimics and inhibitors** | |
| mimics NC sense | UCACAACCUCCUAGAAAGAGUAGA  UCUACUCUUUCUAGGAGGUUGUGA |
| miR-6820-3p mimics | UGUGACUUCUCCCCUGCCACAG  CUGUGGCAGGGGAGAAGUCACA |
| inhibitor NC sense | UCUACUCUUUCUAGGAGGUUGUGA |
| miR-6820-3p inhibitor sense | CUGUGGCAGGGGAGAAGUCACA |
| **ISH Probs** | |
| hsa_circ_0007367 | CAGAAAGCAAGATGACTGAGGACTGA |

**Supplement Table 3. The sequences of oligonucleotides and probes used in this study.**
